# Gene model for the ortholog of *wrd* in *Drosophila ananassae*

**DOI:** 10.17912/micropub.biology.000928

**Published:** 2025-04-01

**Authors:** Megan E. Lawson, Samantha Hoffman, Mikhail Sanu, Daniel Morris, Evan Merkhofer, Stephanie Toering Peters, Nikolaos Tsotakos, Chinmay P. Rele, Laura K. Reed

**Affiliations:** 1 The University of Alabama, Tuscaloosa, AL USA; 2 Penn State Harrisburg, Middletown, PA USA; 3 Mount Saint Mary College, Newburgh, NY USA; 4 Wartburg College, Waverly, IA USA

## Abstract

Gene model for the ortholog of *well-rounded* ( *wrd* ) in the May 2011 (Agencourt dana_caf1/DanaCAF1) Genome Assembly (GenBank Accession: GCA_000005115.1 ) of *Drosophila ananassae* . This ortholog was characterized as part of a developing dataset to study the evolution of the Insulin/insulin-like growth factor signaling pathway (IIS) across the genus *Drosophila* using the Genomics Education Partnership gene annotation protocol for Course-based Undergraduate Research Experiences.

**Figure 1. wrd gene model comparison between Drosophila ananassae and Drosophila melanogaster orthologs:**
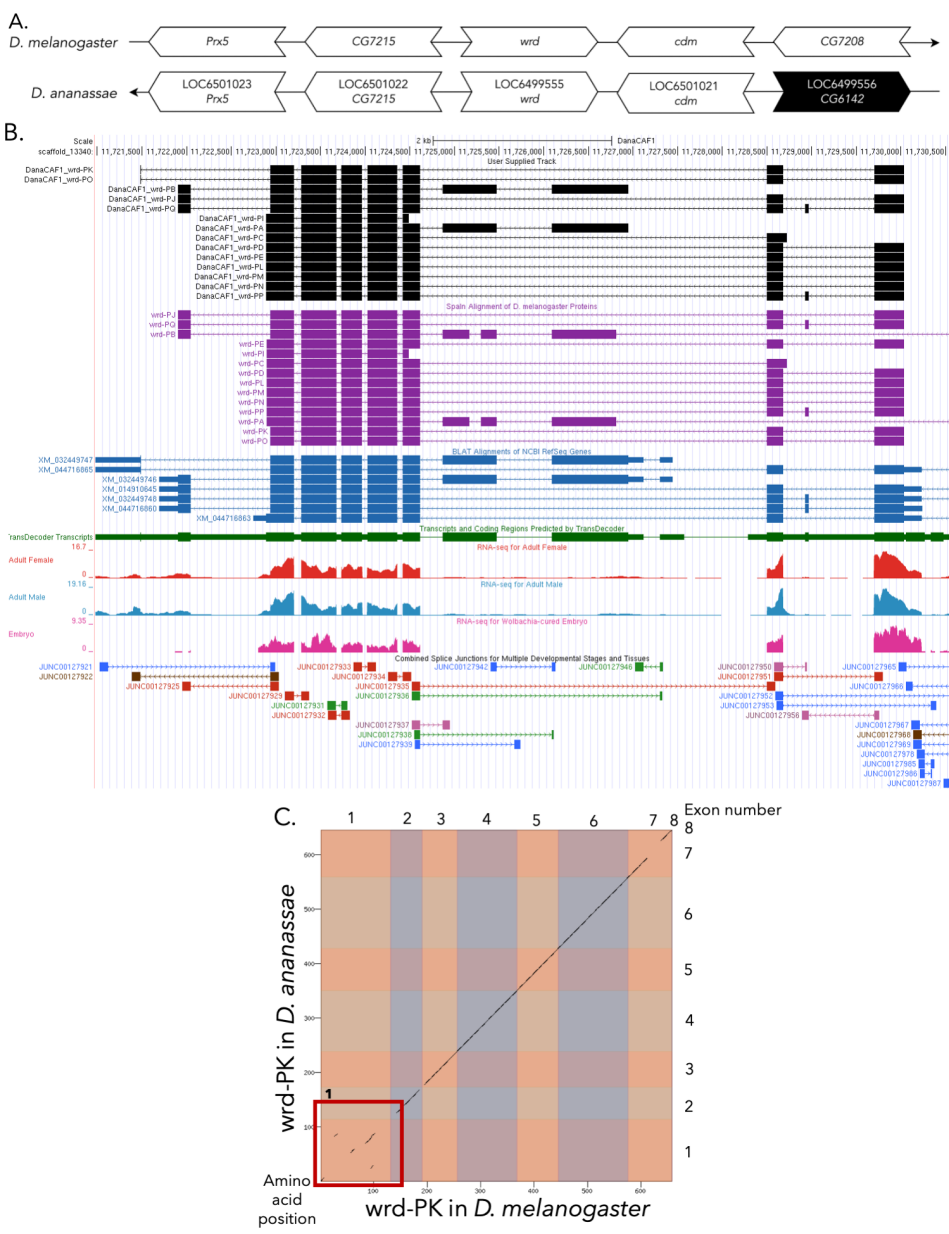
(A) Synteny of genomic neighborhood of *wrd* in *D. melanogaster* and *D. ananassae* . Gene arrows pointing in the same direction as reference gene in both *D. ananassae* and *D. melanogaster* are on the same strand as the reference gene; gene arrows pointing in the opposite direction are on the opposite strand. The thin underlying arrows pointing to the right indicate that *wrd* is on the + strand; arrows pointing to the left indicate that *wrd* is on the – strands. White arrows in *D. ananassae* indicate the locus ID and the orthology to the corresponding gene in *D. melanogaster* and black arrows indicate a non-orthologous gene. The gene names given in the *D. ananassae* gene arrows indicate the orthologous gene in *D. melanogaster* , while the locus identifiers are specific to *D. ananassae* . (B) Gene Model in UCSC Track Hub (Raney *et al.* , 2014): the gene model in *D. ananassae* (black), Spaln of *D. melanogaster* Proteins (purple, alignment of RefSeq proteins from *D. melanogaster* ), BLAT alignments of NCBI RefSeq Genes (blue, alignment of RefSeq genes for *D. ananassae* ), RNA-Seq from adult females (red), adult males (blue), and embryos (pink) (alignment of Illumina RNA-Seq reads from *D. ananassae* ), and Transcripts (green) including coding regions predicted by TransDecoder and Splice Junctions Predicted by regtools using *D. ananassae* RNA-Seq (Gravely *et al.* , 2011; SRP006203 , PRJNA257286 , SRP007906 , PRJNA388952 ). Splice junctions shown have a minimum read-depth of 16 with 10-49, 50-99, 100-499, 500-999, >1000 supporting reads in blue, green, pink, brown, and red respectively. The custom gene model (User Supplied Track) is indicated in black with CDSs depicted with boxes and intron with narrow lines (arrows indicate direction of transcription). (C) Dot Plot of wrd-PK in *D. melanogaster* ( *x* -axis) vs. the orthologous peptide in *D. ananassae* ( *y* -axis). Amino acid number is indicated along the left and bottom; CDS number is indicated along the top and right, and CDSs are also highlighted with alternating colors. Box 1 highlights a region containing some repeats and low sequence similarity.

## Description

**Table:** 

* This article reports a predicted gene model generated by undergraduate work using a structured gene model annotation protocol defined by the Genomics Education Partnership (GEP; thegep.org ) for Course-based Undergraduate Research Experience (CURE). The following information in this box may be repeated in other articles submitted by participants using the same GEP CURE protocol for annotating Drosophila species orthologs of Drosophila melanogaster genes in the insulin signaling pathway. * "In this GEP CURE protocol students use web-based tools to manually annotate genes in non-model *Drosophila* species based on orthology to genes in the well-annotated model organism fruitfly *Drosophila melanogaster* . The GEP uses web-based tools to allow undergraduates to participate in course-based research by generating manual annotations of genes in non-model species (Rele et al., 2023). Computational-based gene predictions in any organism are often improved by careful manual annotation and curation, allowing for more accurate analyses of gene and genome evolution (Mudge and Harrow 2016; Tello-Ruiz et al., 2019). These models of orthologous genes across species, such as the one presented here, then provide a reliable basis for further evolutionary genomic analyses when made available to the scientific community.” (Myers et al., 2024). “The particular gene ortholog described here was characterized as part of a developing dataset to study the evolution of the Insulin/insulin-like growth factor signaling pathway (IIS) across the genus *Drosophila* . The Insulin/insulin-like growth factor signaling pathway (IIS) is a highly conserved signaling pathway in animals and is central to mediating organismal responses to nutrients (Hietakangas and Cohen 2009; Grewal 2009).” (Myers et al., 2024). “ *D* . *ananassae* (NCBI:txid7217) is part of the *melanogaster* species group within the subgenus *Sophophora* of the genus *Drosophila* (Sturtevant 1939; Bock and Wheeler 1972). It was first described by Doeschall (1858). *D. ananassae* is circumtropical (Markow and O'Grady 2005; https://www.taxodros.uzh.ch , accessed 1 Feb 2023), and often associated with human settlement (Singh 2010). It has been extensively studied as a model for its cytogenetic and genetic characteristics, and in experimental evolution (Kikkawa 1938; Singh and Yadav 2015).” (Lawson et al., 2024).

The model presented here is the ortholog of *wrd* in the May 2011 (Agencourt dana_caf1/DanaCAF1) assembly of *D. ananassae* ( GCA_000005115.1 – Drosophila 12 Genomes Consortium et al., 2007) and corresponds to the Gnomon Peptide ID ( XP_032305638.1 ) predicted model in *D. ananassae* ( LOC6499555 ) *.* This gene model is based on RNA-Seq data from *D. ananassae* (Gravely et al *.* , 2011; SRP006203 , PRJNA257286 , SRP007906 , PRJNA388952 ) and the *wrd* ( GCA_000001215.4 ) in *D. melanogaster* from FB2022_03 (Larkin et al *.,* 2021; Gramates et al., 2022; Jenkins et al., 2022).

The *well-rounded* ( *wrd* ) gene was first identified in a gain-of-function screen for molecules that regulate synaptic development in the neuromuscular junction (NMJ) in *Drosophila melanogaster* (Viquez et al., 2006). Its gene product is one of the two B' regulatory subunits of protein phosphatase 2A (PP2A), which interacts with liprin-α to stabilize specification of the active zone, a site in the axonal plasma membrane that regulates endo- and exocytosis of synaptic vesicles (Li et al., 2014). Another interacting partner of wrd is S6K, an important kinase in the insulin/TOR signaling pathway that is targeted for dephosphorylation by the PP2A holoenzyme through its molecular interaction with wrd (Hahn et al., 2010). Loss-of-function *wrd* mutants are viable and have fewer synaptic boutons that are larger and have a smooth round contour, a phenotype which gave the gene its name (Viquez et al., 2006). Knockout flies for the *wrd* B' subunit of PP2A are lean, have a reduced lifespan, and elevated insulin signaling (Hahn et al., 2010), with tissue specificity fine-tuned by cyclin G (Fischer et al., 2016).

***Synteny***

*wrd* occurs on chromosome 3L in *D. melanogaster* and is flanked upstream by *CG7215* and *Peroxiredoxin 5* ( *Prx5 )* , and downstream by *cadmus* ( *cdm )* and *CG7208 .* We determined that the putative ortholog of *wrd* is found on scaffold 13340 ( CH902617.1 ) in *D. ananassae* with LOC6499555 ( XP_032305638.1 ) (via *tblastn* search with an e-value of 0.0 and percent identity of 88.81%). The *wrd* ortholog is flanked upstream by LOC6501022 ( XP_001954393.1 ) and LOC6501023 ( XP_001954394.2 ) which correspond to *CG7215* and *Prx5* in *D. melanogaster* with e-values of 7e-78 and 3e-127, respectively, and percent identities 82.31% and 93.68%, respectively, as determined by *blastp* . The *wrd* ortholog is flanked downstream by LOC6501021 ( XP_001954390.2 ) and LOC6499556 ( XP_001954389.1 ) which correspond to *cdm* and *CG6142* in *D. melanogaster* with e-values of 0.0 and 0.0, respectively, and percent identities of 87.44% and 88.64%, respectively, as determined by *blastp* ( Figure 1A, Altschul et al *.* , 1990). We believe this is the correct ortholog assignment for *wrd* in *D. ananassae* because synteny is well-conserved, with only one gene in the genomic neighborhood being non-orthologous, and because all of the BLAST searches used to determine orthology were very high quality.

***Protein Model***

*wrd* in *D. ananassae* has nine unique protein-coding isoforms ( Figure 1B ). The protein isoform encoded by mRNAs *wrd-RD, wrd-RE, wrd-RM, wrd-RL* , and *wrd-RN* (which differ in their UTRs) contains seven CDSs. The protein isoform encoded by mRNAs *wrd-RK* and *wrd-RO* contain eight protein-coding CDSs. Isoform wrd-PJ, is encoded by *wrd-RJ* which contains eight CDSs. Protein isoform wrd-PI is encoded by five CDSs. Isoform wrd-PQ is encoded by nine CDSs. Isoform wrd-PP is encoded by eight CDSs. Isoform wrd-PC is encoded by six CDSs. Isoform wrd-PB is encoded by eight CDSs. Isoform wrd-PA is encoded by seven CDSs. This isoform strucutre is the same relative to the ortholog in *D. melanogaster* , which also has 9 unique protein-coding isoforms. All of the *D. melanogaster* isoforms have the same number of CDSs as their corresponding ortholog in *D. ananassae.* The amino acid sequence of *wrd* in *D. ananassae* has 83.0% identity with the *wrd* in *D. melanogaster* as determined by *blastp* ( Figure 1C ). The coordinates of the curated gene models can be found in NCBI at GenBank/BankIt using the accessions BK064428 , BK064429 , BK064430 , BK064431 , BK064432 , BK064433 , BK064434 , BK064435 , BK064436 , BK064437 , BK064438 , BK064439 , BK064440 , and BK064441 . These data are also available in Extended Data files below, which are archived in CaltechData.

## Methods

Detailed methods including algorithms, database versions, and citations for the complete annotation process can be found in Rele et al. (2023). Briefly, students use the GEP instance of the UCSC Genome Browser v.435 ( https://gander.wustl.edu ; Kent WJ et al., 2002; Navarro Gonzalez et al., 2021) to examine the genomic neighborhood of their reference IIS gene in the *D. melanogaster* genome assembly (Aug. 2014; BDGP Release 6 + ISO1 MT/dm6). Students then retrieve the protein sequence for the *D. melanogaster* reference gene for a given isoform and run it using *tblastn* against their target *Drosophila* species genome assembly on the NCBI BLAST server ( https://blast.ncbi.nlm.nih.gov/Blast.cgi ; Altschul et al., 1990) to identify potential orthologs. To validate the potential ortholog, students compare the local genomic neighborhood of their potential ortholog with the genomic neighborhood of their reference gene in *D. melanogaster* . This local synteny analysis includes at minimum the two upstream and downstream genes relative to their putative ortholog. They also explore other sets of genomic evidence using multiple alignment tracks in the Genome Browser, including BLAT alignments of RefSeq Genes, Spaln alignment of *D. melanogaster* proteins, multiple gene prediction tracks (e.g., GeMoMa, Geneid, Augustus), and modENCODE RNA-Seq from the target species. Detailed explanation of how these lines of genomic evidenced are leveraged by students in gene model development are described in Rele et al. (2023). Genomic structure information (e.g., CDSs, intron-exon number and boundaries, number of isoforms) for the *D. melanogaster* reference gene is retrieved through the Gene Record Finder ( https://gander.wustl.edu/~wilson/dmelgenerecord/index.html ; Rele et al *.,* 2023). Approximate splice sites within the target gene are determined using *tblastn* using the CDSs from the *D. melanogaste* r reference gene. Coordinates of CDSs are then refined by examining aligned modENCODE RNA-Seq data, and by applying paradigms of molecular biology such as identifying canonical splice site sequences and ensuring the maintenance of an open reading frame across hypothesized splice sites. Students then confirm the biological validity of their target gene model using the Gene Model Checker ( https://gander.wustl.edu/~wilson/dmelgenerecord/index.html ; Rele et al., 2023), which compares the structure and translated sequence from their hypothesized target gene model against the *D. melanogaster* reference gene model. At least two independent models for a gene are generated by students under mentorship of their faculty course instructors. Those models are then reconciled by a third independent researcher mentored by the project leaders to produce the final model. Note: comparison of 5' and 3' UTR sequence information is not included in this GEP CURE protocol.

## Data Availability

Description: GFF, FASTA, and PEP of the model. Resource Type: Model. DOI: https://doi.org/10.22002/9mj0d-yn206
